# Sea Surface Imaging with a Shortened Delayed-Dechirp Process of Airborne FMCW SAR for Ocean Monitoring on Emergency

**DOI:** 10.3390/s20247310

**Published:** 2020-12-19

**Authors:** Ji-hwan Hwang, Duk-jin Kim

**Affiliations:** 1 Research Institute of Basic Sciences, Seoul National University, Seoul 88026, Korea; hwang1651@snu.ac.kr; 2 School of Earth and Environmental Science, Seoul National University, Seoul 88026, Korea

**Keywords:** sea surface imaging, airborne FMCW SAR, delayed-dechirp process, incidence angle

## Abstract

A sea surface imaging technique for an emergency response using a ready-made frequency modulated continuous wave–synthetic aperture radar (FMCW SAR) system and its experimental results are described in this paper. The optimal range of radiowave incidence angle for sea surface imaging was analyzed by a theoretical scattering model and measurement data, and it was properly applied to the FMCW SAR system by readjusting the delayed-dechirp process. Raw data acquired through flight experiments were reconstructed to SAR image by the range-doppler algorithm. To verify the performance of the reconstructed sea surface image, dual-channel images collected by the configuration of the along-track interferometry were used, and then performance indicators such as signal attenuation, coherence, and phase difference were analyzed. Through this experimental study, it was confirmed that the ready-made FMCW SAR system without a function of the incident angle control can also conduct limited missions for maritime observation. It is possible to be an alternative resource for emergency response, in which the cases are requiring urgent maritime disaster detection and analysis.

## 1. Introduction

Through ocean observation technology using a synthetic aperture radar based on microwave remote sensing, it is possible to detect and analyze over a wide area the occurrence of emergencies or disasters such as a hurricane, tsunami, sea pollution, maritime accident, and it is easy to generate various data to inform a quick response to the situation [1,2,3]. However, a high-resolution SAR system for sea surface imaging might be a high-cost system in terms of required performance and utilization because it needs a precise 3D gimbal system to control a radiowave incident angle in a low range and a high-performance platform flying at high altitude to get a wide swath width. In particular, a 3D gimbal system must include specific functions to compensate for aircraft motion and vibration such as a yaw canceling function, and to control the incident angle in an elevation direction as well [4]. However, we can consider an alternative way to observe the sea surface with a ready-made airborne synthetic aperture radar (SAR) system, which can replace the cases where the high-performance systems are not available.

Conducting an ocean monitoring mission with a ready-made SAR system customized for ground observation can be a cost-effective alternative in an emergency, in which the cases are requiring urgent maritime disaster detection and analysis, even though it has limited performance. The airborne frequency modulated continuous wave–synthetic aperture radar (FMCW SAR) system used in this study has a 2D gimbal system, which only has the yaw canceling function without an elevation angle control, and it can be easily installed at various platforms such as a light airplane or ground vehicle [5]. On the other hand, it is difficult to imaging the sea surface in a focused SAR image because of the fixed incident angle of the radar beam. This is due to the microwave polarimetric responses, in which the backscattering from the randomly rough sea surface decreases rapidly as the incident angle increases [6,7]. Additionally, it is easy to see that various types of water surface in a focused SAR image are represented in dark color with a very low signal level. Therefore, in order to accurately image the dark water surface with a low signal level, it is essential to obtain a stable received signal measured through a low range of incident angle control and also stably operate the SAR system reflecting this condition. However, this compact cost-effective SAR system customized for ground observation does not have a function of incident angle control so that it should have adopted an indirect method with FMCW radar signal processing, the so-called delayed-dechirp [8]. This process can control a starting point of a sampled received signal. In addition, using this point, the incident angle control to receive the backscattered signal from the randomly rough sea surface is tried indirectly and the results of the sea surface imaging are validated by flight experiment.

FMCW SAR system has several signal characteristics that are distinguished from the pulse signal-based system. Among them, the delayed-dechirp process that down converts the beat frequency to a specific intermediate frequency (IF) makes it possible to implement a compact cost-effective radar system. A specific range of sea clutter can be selectively received by this delayed-dechirp process, and it can be an alternative to control the incident angle of a ready-made SAR system with a fixed incident angle [9,10,11]. The airborne FMCW SAR system (NanoSAR-B, ImSAR Co.,Utah, UT, USA) used in this study is a compact and lightweight system with a fixed incident angle of 45°, and an optimized swath width based on the delayed-dechirp process is automatically set [5]. To apply this to maritime observation missions, the swath width is automatically set based on a specific flight altitude, and the incident angle is manually reset to the near-nadir look angle by the delayed-dechirp process. In this way, the sea clutter signal is collected and imaged.

In this study, polarimetric responses of the sea surface according to the change of the incident angle are analyzed based on the theoretical scattering model [6] and observation data, and the range of incident angle suitable for performing the maritime observation mission is applied to the airborne FMCW SAR system. Then, this technique for imaging the sea surface is verified by the flight experiment. The received signals at near-nadir look angle are processed by range doppler algorithm. Using the focused SAR images, the feasibility of using the alternative approach for the maritime observation mission is verified. The polarimetric responses of the sea surface in each incident angle and the readjusted delayed-dechirp process are presented in Section 2 and Section 3, and the flight experiments applied the optimal incident angle setting and their results are presented in Section 4 and Section 5, respectively.

## 2. Sea Clutter

**Figure 1:**
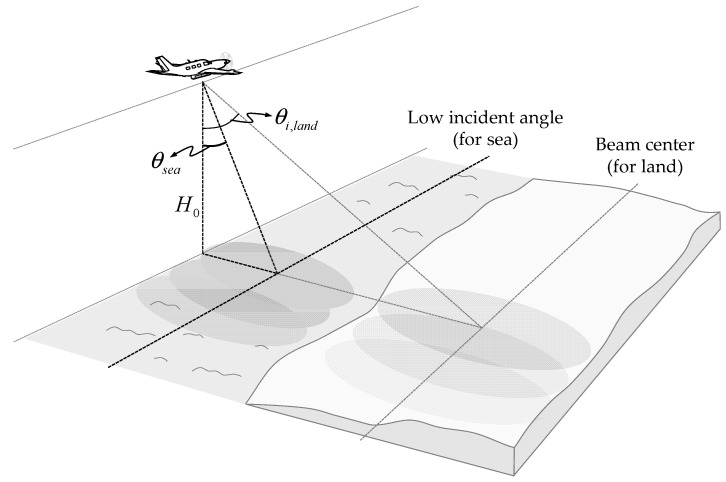
Geometry of the airborne frequency modulated continuous wave–synthetic aperture radar (FMCW SAR) system for a maritime observation mission.

Airborne SAR systems for a ground observation have generally been optimized with a focus on small and lightweight configurations, high-resolution image quality, and performance with wide swath width [9,10]. As shown in Figure 1, in the case of a small and lightweight airborne SAR system with improved operational convenience, SAR images can be restored by collecting the signals backscattered from the ground or sea surface fixed at a specific angle of incidence. However, since the incidence angle and swath width for a maritime observation are different from those optimized for ground observation, it is essential to change the settings and analyze the characteristics for stable signal reception. The sea clutter signal has a characteristic where the received signal level rapidly decreases as the incident angle increases compared to the ground observation signals. The effect of such sea clutter signal characteristics on data acquisition and sea surface imaging using an airborne SAR system is analyzed using a theoretical model and observation data. The theoretical backscatters of the sea surface can be analyzed based on the well known integral equation scattering model (IEM) [6,7]. By comparing and analyzing ocean observation data using a self-manufactured scatterometer system, the change in the backscattering coefficient according to each incident angle is analyzed.

**Figure 2:**
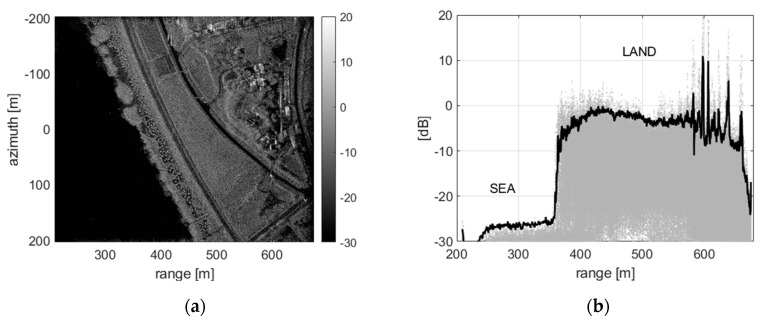
Example of a SAR image including the coastal area: it was acquired by the X-band airborne FMCW SAR system applying an optimal setup for ground observation. (**a**) is a reconstructed SAR image, and (**b**) is its range profile of signal intensity.

Figure 2 shows the SAR image of an area adjacent to the sea, reconstructed by the original data of an airborne FMCW SAR system optimized for ground observation. To acquire raw data, the parameters of X-band operating frequency, at a fixed incident angle of 45°, and a flight altitude of about 430 m, were applied, and it was reconstructed as a SAR image with a swath width of about 500 m and a synthetic aperture length of 400 m. The dark area on the left in Figure 2a represents a calm sea surface, which is in contrast to the brightly displayed surface in the SAR image, and the difference is about more than 25 dB as shown in Figure 2b. The low signal level of sea clutter identified through the reconstructed SAR image makes it difficult to image the sea surface for the maritime observation mission. Therefore, a property of the polarimetric backscattering from the sea surface should be first analyzed by IEM, and to increase the sea clutter signal to a signal region capable of imaging the sea surface, a suitable system setup to adjust the incident angle should be reflected too.

First, a range of the incident angle that can restore the sea surface is analyzed using the theoretical scattering model IEM. The range of the incident angle, in which the sea clutter signal level maintains above the threshold for SAR image restoration, can be roughly analyzed and estimated using the calculation result of IEM. Although the actual sea clutter cannot be completely reproduced with only IEM, it can provide sufficiently predictable information on the change of the sea clutter for each angle of incidence. The IEM applied to estimate the scattering property of the sea surface can simulate the polarimetric responses by using the radar operating frequency, polarization, incidence angle, permittivity, and roughness (e.g., RMS height, correlation length) of the sea surface as input variables [6,7]. Sea surface with a specific roughness can be expressed as RMS height (ks) and correlation length (kl) using a PM spectrum reflecting the wave height proportional to wind strength [12,13,14], and its polarimetric response can be calculated by applying the relative permittivity of seawater (εr = 81-j698.8).

**Figure 3:**
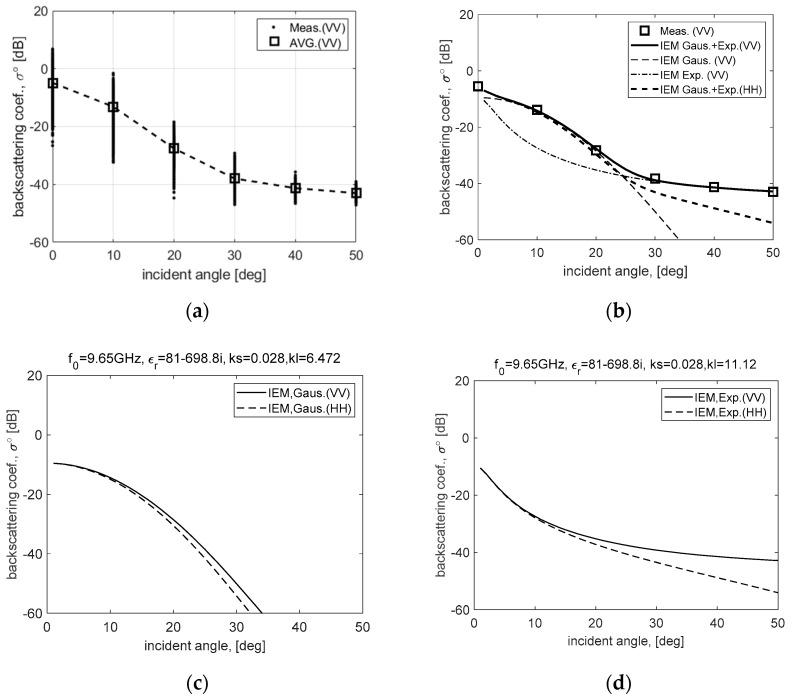
Backscattering coefficients from the sea surface simulated by a theoretical scattering model of an integral equation scattering model (IEM): (**a**) is in situ data measured by the FMCW signal-based scatterometer at the Ieodo ocean research station (Korea Hydrographic and Oceanographic Agency); (**b**) is a composite polarimetric response with two-scale (**c**) Gaussian- and (**d**) exponential-correlated surfaces, (for windy sea surface within about 2 m/s to 4.5 m/s).

Figure 3 is an example to analyze the polarimetric responses of the sea surface by comparing the theoretical scattering model and observation data, and it shows an attenuation property of the sea clutter signal according to the incident angle using a composite surface. Figure 3a is the calibrated result of the observation data measured at Ieodo ocean research station using a FMCW signal-based scatterometer system. These observation data show the backscattering coefficients calibrated for the received signal of the FMCW signal-based scatterometer system and is the calibrated result by applying the differential Mueller matrix calibration technique (DMMCT) [15]. In order to calibrate the observation data of the single polarization (VV-pol.), the DMMCT based on the full polarization was partially modified, and it conducted the calibration procedures including an absolute calibration and a relative calibration process. The absolute calibration process is comparing the theoretical polarimetric response to the measured data of a reference target such as a metal sphere, and the relative calibration process is compensating the distorted responses of differential targets in antenna footprint by antenna radiation pattern. This is the result obtained by simultaneously performing calibration processes using the information of the Tx/Rx antenna radiation pattern accurately measured at 1° resolution.

Figure 3b shows the polarimetric responses of the composite sea surface with two types of wave spectrum (Figure 3c,d) simulated using the IEM scattering model and compares the observation data in Figure 3a. Here, the observation data were measured on the windy sea surface of 2 to 4.5 m/s, and a capillary wave of sea surface having a roughness corresponding to them was generated and applied to the IEM scattering model. Figure 3c,d are the simulation results using the IEM scattering model, the specific roughness for Gaussian- and exponential-correlated surface were applied such as ks = 0.028, kl = 6.472 and ks = 0.028, kl = 11.12, respectively.

As shown in Figure 3b, it can be confirmed that the polarimetric responses of the sea surface (IEM Gaus. + Exp.), in which two types of wave spectrum are synthesized [7], correspond very well to the observation data using vertical polarization data, and the response of horizontal polarization (HH-pol.) can be also predicted through the calculation results of the IEM scattering model. Moreover, the polarimetric responses of the windy sea surface analyzed using observation data and simulation results show a signal attenuation of about 25 dB in the incident angle near 20° for both vertical and horizontal polarization. It can be easily understood from the SAR image in Figure 2 that the sea clutter signal must keep a higher signal level than the noise floor in the reconstructed SAR image. Therefore, SAR raw data for sea surface imaging must be collected at an incident angle within 20° at least, and system settings must be properly reflected. Here, a predicted threshold of the observation angle for sea surface imaging is an analysis result dependent on the observation data of a windy sea surface of 2 to 4.5 m/s and can be used as a guideline for the operation of an airborne SAR system.

## 3. FMCW SAR with a Low Incident Angle

The next step is to collect the raw data of low incident angle using the ready-made FMCW SAR system that does not have a function to adjust the incidence angle in an elevation angle direction. Fortunately, the FMCW-based radar system has a function of delayed-dechirp process, which can be used to collect raw data in a specific incidence angle. A method of collecting the raw data of sea clutter at a low incidence angle using a shortened delayed-dechirp process is described in this section.

Equations (1)~(4) are FMCW SAR signal models, which represent the transmitted signal(*s_0_ (t)*), the delayed transmitted signal(*s_0,d_ (t)*), the received signal(*s_r_ (t)*) including the target delay time (τ), and the received signal(*s_dc_ (t)*) frequency down converted by the delayed transmitted signal(*s_0,d_ (t)*), respectively [8,10]. The range profile of the target located at a specific distance (*R*) is analyzed from the intermediate frequency (IF) received signal obtained by frequency down-conversion using the delayed transmitted signal. The IF received signal can be sampled at a specific sampling frequency, and a maximum range of the beat frequency spectrum transformed by Fourier transform is limited to half the sampling frequency by the Nyquist sampling theorem. The beat frequency spectrum is converted into a range profile by beat frequency to a range ratio (*fb/R = 2Kr/c*: *Kr* chirp rate, *c* light speed), and analyzing the range profile from the dechirp-delay time (*d*) to targets is possible:

(1) $$s_{0}\left(t\right)\;=\;\exp\left\{-j2\pi \left(f_{0}t\;+\;\frac{K_{r}}{2}t^{2}\right)\right\}$$

(2) $$s_{0,d}\left(t\right)\;=\exp\left\{j2\pi \left(f_{0}\left(t-d\right)\;+\;\frac{K_{r}}{2}{\left(t-d\right)}^{2}\right)\right\}$$

(3) $$s_{r}\left(t,u\right)=\exp\left\{-j2\pi \left(f_{0}\left(t-\tau \left(u\right)\right)+\frac{K_{r}}{2}{\left(t-\tau \left(u\right)\right)}^{2}\right)\right\}$$

(4) $$s_{dc}\left(t,u\right)\;=\;s_{r}\left(t,u\right)\;\times \;s_{0,d}^{*}\left(t,u\right)\;=\;\exp\left\{j2\pi \left(f_{0}\;+\;K_{r}t\right)\left(\tau \left(u\right)-d\right)-j\pi K_{r}\left(\tau^{2}\left(u\right)-d^{2}\right)\right\}$$

**Figure 4:**
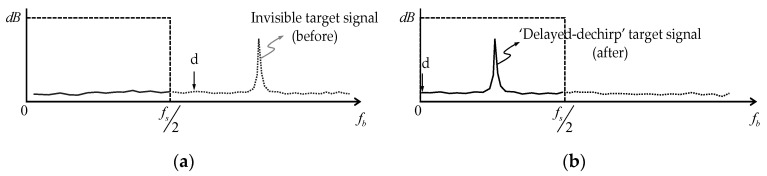
Beat frequency spectrum readjusted by the delayed-dechirp process: (**a**,**b**) is before and after applying the delayed-dechirp process, respectively.

In the case of an airborne FMCW SAR system, because the target of interest does not exist within the flight altitude at least, the range profile containing unnecessary information results in the inefficient operation of system resources, and the delayed-dechirp process can be used to solve such problems. It uses the delayed transmission signal of Equation (2) including a specific delay time (*d*) instead of the reference transmission signal of Equation (1) to convert the received signal of Equation (3) to Equation (4). Through frequency down converting, as shown in Figure 4, it is possible to observe a distant target outside the existing observation range even with the same sampling frequency.

It is a technique widely used in FMCW SAR systems as a method of radar signal processing for long-range detection out of the maximum range limited to half of the sampling frequency, as described above [8]. The airborne FMCW SAR system used in this study also adopts this type of signal processing technology. Fortunately, it has the system graphic user interface including a subfunction called the ‘center slant range offset’, which provides users with stepwise delayed dechirp functions. The SAR raw data acquired in the low incidence angle for the maritime observation mission can be proceeded with the following concept by utilizing the delayed dechirp function provided by the existing system.

**Figure 5:**
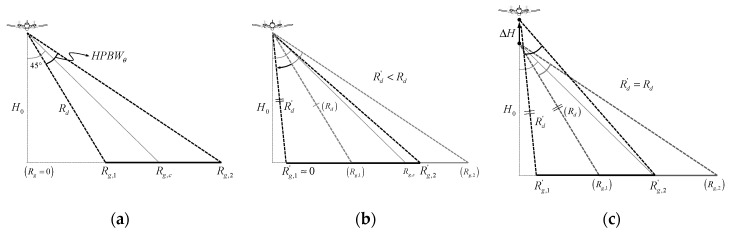
Geometry of the delayed-dechirp process: (**a**) is the automatic setup referred to as a beam center of the fixed incident angle, and (**b**,**c**) are two options of manual setup to adjust the incident angle.

Figure 5 shows each experimental condition to which the delayed-dechirp process was applied. Figure 5a is the case where the SAR system setting optimized for the ground observation mission is applied, Figure 5b,c are the cases in which the adjusted dechirp-delay(*d′ = 2R_d_′/c*) is applied to acquire SAR raw data at the low incidence angle. The optimal dechirp-delay setting for the ground observation mission is as shown in Figure 5a, the value automatically set based on the half-power beam width is applied, while Figure 5b shows the case where the automatically set dechirp-delay(*d*) is replaced with the adjusted dechirp-delay (*d′*), which is smaller than the automatically set original value. Therefore, it is possible to acquire the raw data within a low incident angle only by adjusting the dechirp-delay in the available range (*d > d′ ≥ 2H_0_/c*), and the dechirp-delay can be adjusted up to a delay time corresponding to the flight altitude (*H_0_*). This means that it is theoretically possible to collect the raw data within the maximum detectable range from the vertically incident area. Moreover, because the resolution of the SAR image rapidly deteriorates as it approaches a nadir direction, it is needed to analyze in a beam pattern and resolution point of view.

Additionally, Figure 5c is an option that can be selected when the adjustment of the dechirp-delay of the existing airborne FMCW SAR system is limited. It is possible to collect raw data in a low incidence angle range by flying higher than the preset flight altitude (*H*_0_). However, this way requires an enforced mission plan establishment for sea surface imaging in terms of system operation, and there is a disadvantage that the system setting becomes complicated accordingly. In addition, after collecting the raw data, an additional data conversion process was required for image reconstruction. Nevertheless, in terms of performing maritime observation missions using the ready-made FMCW SAR system, the approaches in Figure 5b,c are still effective alternatives.

To apply the above system configuration to an existing FMCW SAR system, Tx/Rx antennas with a wide beamwidth are required. In other words, the raw data acquired by the antenna with a fixed incident angle physically and the readjusted dechirp-delay forcibly become data collected in the area off the center of the beam pattern in the elevation direction. Therefore, for a more stable SAR, raw data collection in a wide incidence angle range, the beam pattern of the Tx/Rx antennas should have a sufficiently wide beamwidth in the elevation direction, whereas the azimuth beam pattern should have a relatively narrow beamwidth in terms of antenna gain and SAR image resolution.

**Figure 6:**
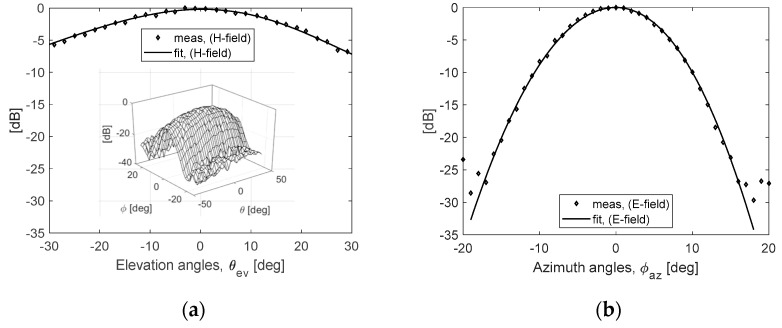
Measured Tx/Rx antenna beam pattern for the airborne FMCW SAR system: (**a**) shows the elevation cut (half-power beamwidth 37.9°) and 3D pattern, and (**b**) is the azimuth cut (half-power beamwidth 11.4°).

Figure 6 is the measurement result of antenna radiation pattern of the airborne FMCW SAR system applied in this study, and the half-power beamwidths in the elevation and azimuth directions were measured as 37.9° and 11.4°, respectively. Figure 6a shows the measurement result of a 3D beam having a fan-shaped beam pattern that is wide in the elevation direction and narrow in the azimuth direction. The signal attenuation characteristic of the received signal at the low incident angle is obtained by multiplying the distance attenuation (*1/R^2^*) component, the antenna beam pattern (*H(θ_ev_))* component, and the target’s backscattering coefficients (*σ°(θ_inc_)*), as shown in Equation (5). Using this, it is possible to easily predict a change in the signal attenuation according to the change in the angle of incidence (*θ_inc_*) or the ground range (*R_g_*):

(5) Atten(θinc) µ {σ°(θinc)} × {1R(θinc)} × {H(θinc−45°)}

**Figure 7:**
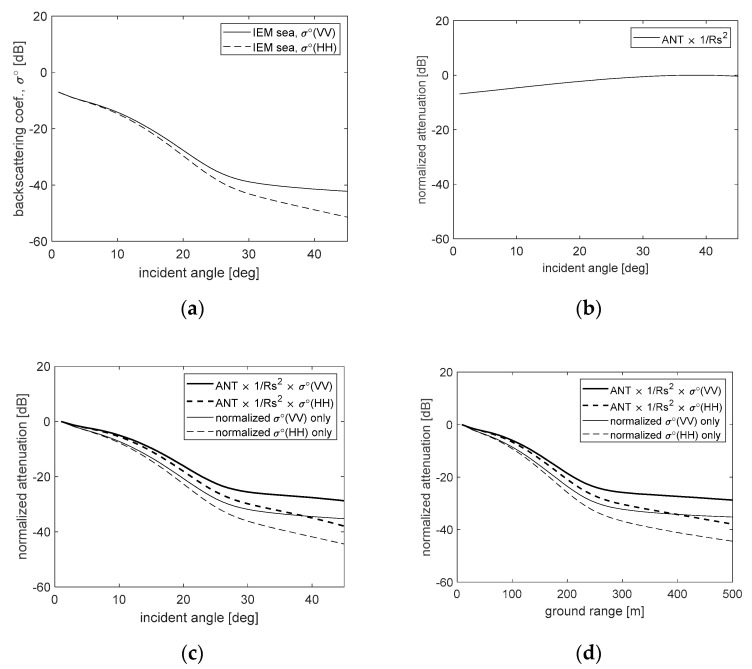
The property of the attenuation of sea clutter signal in each incident angle: (**a**) is the backscattering coefficients calculated by IEM; (**b**) is a normalized attenuation calculated by the antenna beam pattern and slant range; and (**c**,**d**) are the normalized total attenuations represented by the incident angle and ground range, respectively.

For example, the signal attenuation can be analyzed by reflecting the simulated backscattering coefficients of the sea surface (Figure 3b), the measured antenna beam pattern (Figure 6a), a flight altitude of 500 m and a fixed incident angle of 45°, as shown in Figure 7. Figure 7a is an example of the backscattering coefficients of sea surface analyzed previously and Figure 7b shows the normalized attenuation obtained by multiplying the elevation beam pattern and the distance attenuation. Due to the influence of the antenna beam pattern, the normalized attenuation is minimized at an incidence angle of 39° ahead of the beam center and shows an attenuation of about −7 dB near the nadir angle. Moreover, Figure 7c,d are the normalized total attenuation predicted by the incident angle and the ground range, respectively. The maximum area that can reconstruct the sea surface image based on −25 dB signal attenuation compared to the maximum received signal is analyzed, with an incident angle of about 30° of and a ground range of about 288 m. By the property of the attenuation of Figure 7b, the total attenuation of the sea clutter signal due to a change of the incident angle or the ground range is slightly improved, about 4.6 dB at the incident angle of 20° (*R_g_* ≈ 182 m) and about 7 dB at the incident angle of 30° (*R_g_* ≈ 288 m).

Then, the theoretical azimuth resolution of the airborne FMCW SAR system adopted in this study can be analyzed from the measurement result of the azimuth beam pattern in Figure 6b. When the maximum effective length of the synthetic aperture for image reconstruction is defined as the half power beam width (11.4°) of the Tx/Rx antenna, the theoretical azimuth resolution (*ΔAz = 2π/Ω, Ω* Doppler frequency bandwidth) is about 7.5 cm [16].

## 4. Flight Experiment

A field campaign was conducted in a coastal area (Goheung county, South Korea) to verify the performance of sea surface imaging using the ready-made FMCW SAR system without a function of incident angle control. The specifications of the FMCW SAR system applied to the flight experiment are as shown in Table 1 below, and it has a system default setting for acquiring dual-channel images with an operating frequency of the X-band and 0.3 m resolution [5].

**Figure 8:**
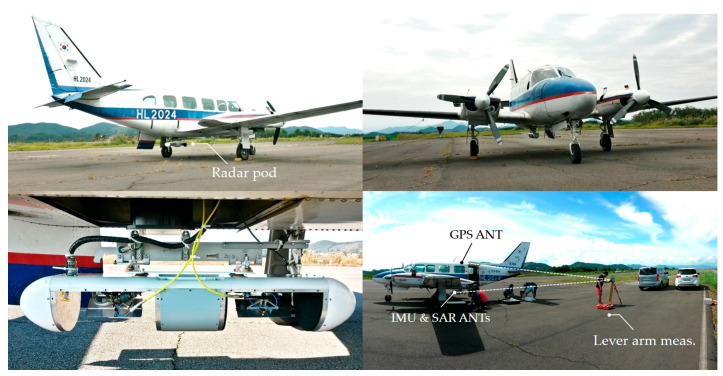
Overview of the airborne FMCW SAR system: radar pod was mounted at the bottom of the fuselage of PIPER PA31-350 and Tx/Rx antennas of NanoSAR-B were installed for left-looking as a default setup. The lever arm was measured with a total station, then its spatial coordinate was entered into NanoSAR-B as a system parameter and used for SAR image focusing.

Figure 8 shows the preparation process of installing the radar system and measuring the lever arm for the flight experiment. The radar pod was installed under the fuselage of the aircraft (PIPER PA31-350) in consideration of the flight safety and observation convenience, and, to receive a GPS signal well, a GPS antenna located at the top side of the fuselage was used. Here, an accurate lever arm measurement is required to correct the GPS information collected at a location separated from the radar pod. Using a total station and a 3D scanner, the distance among the GPS antenna, the IMU (inertial measurement unit) sensor, the Tx/Rx antennas, and the gimbals inside the radar pod were accurately measured. The spatial coordinate information of the lever arm is applied to the precise coordinate conversion process for the motion error compensation with GPS and IMU data [9]. The raw data acquired through flight experiments are focused by range-doppler algorithm (RDA) [11,17]. The reconstructed SAR images were calibrated by the external calibration procedure using a calibration target such as a trihedral corner reflector [18], and it was conducted under the normal setting for a look angle referred to beam center. To verify the performance of the sea surface imaging technique, which acquires raw data at a low incident angle, dual-channel SAR images acquired through the configuration of along-track interferometry were used. Since it is difficult to perform the field campaigns for system calibration and validation above the sea surface, the coherence and phase difference of the dual-channel images were applied as a performance indicator to indirectly measure the accuracy and stability of complex signals in dual-SAR images. Moreover, the range profile of the signal attenuation is also used as a tool to determine the threshold for sea surface imaging.

**Figure 9:**
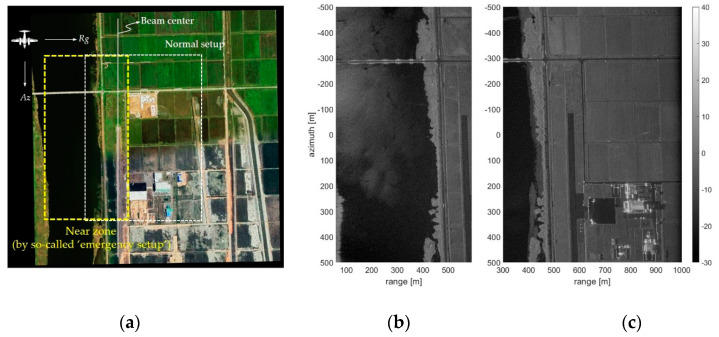
Comparison result of the flight experiment with the different setups: (**a**) is an optical image of estuary terrain; (**b**) shows the near zone SAR image reconstructed by the shortened delayed-dechirp process of airborne FMCW SAR system, we call it an ‘emergency setup’; and (**c**) shows the SAR image reconstructed by the normal setup as a default, respectively.

Figure 9 shows the comparison result of an extended capability applying the shortened delayed-dechirp process. As described above, the so-called ‘emergency setup’ applying the shortened delayed-dechirp process shows that it is possible to acquire a near zone SAR image with a low incident angle range compared to the normal setup based on the beam center. To verify the proposed technique for acquiring the raw data at a low incident angle, the test area was selected as an estuary area adjacent to the sea. Since this area includes a river and a plain, it was easy to compare the change of incidence angle in the reconstructed SAR images. This area was also used as a test area for the system calibration of the normal setup, in which the trihedral corner reflectors as a calibration target were located at a ground range of about 600 m beside the runway, as shown in Figure 9c. Here, the flight path was set in a direction parallel to the runway so that the river and plain were distinguished by the incident angle. The shortened dechirp-delay (*d′ ≈ 2H*_0_*/c*) and the normal dechirp-delay (*d ≈ 2R_d_/c*) were set to adjust the incident angle of the airborne FMCW SAR system, as follows. The flight altitude(*H*_0_) is about 500 m, and the dechirp-delays for the different setups are about *d′* = 3.3 µs and *d* = 3.9 µs. In addition, the ranges of incident angle were adjusted to about 3.5~49° and 30~63°, respectively (see Figure 5).

**Figure 10:**
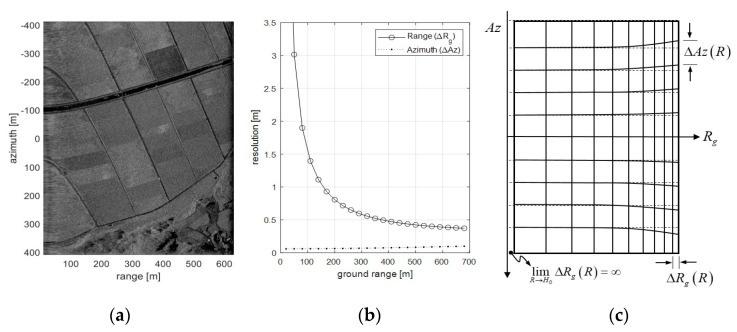
Resolution variation of the SAR image reconstructed with the raw data acquired within the range of low incident angle: (**a**) is an SAR image reconstructed by the range doppler algorithm; (**b**) is a calculated resolution; and (**c**) is a resolution grid depicted exaggeratedly for the near zone SAR image.

Figure 10 is a SAR image obtained from experimental conditions reflecting the readjusted delayed-dechirp process as mentioned before, and is the analysis result of the resolution in SAR image. Figure 10a is a SAR image observed in a flat land, and it has a characteristic that a start point of the ground range is almost zero, because of the dechirp-delay set near the flight altitude (*H*_0_ = 500 m). Changes in the resolution of the SAR image projected to the ground are as shown in Figure 10b. It can be confirmed that the range resolution is rapidly increased in the vicinity of the nadir angle, whereas the azimuth resolution is slightly improved as the ground range becomes closer. This is because the range resolution (*ΔR/cos(θ_i_)*) is strongly influenced by the incident angle and the Doppler frequency bandwidth slightly increases as the ground range gets closer. Therefore, the shortened dechirp-delay setting applied for the sea surface imaging enables the collection of raw data in the near zone while it has a non-uniform resolution grid characteristic as shown in Figure 10c. The range resolution is about 0.42 m based on the beam center (*R_g_* = 500 m), and it can be seen that it increases more than three times to 1.5 m at the ground range of 100 m.

It was consequently confirmed that the shortened delayed-dechirp process proposed in this study can obtain the raw data within the low incident angle and reconstruct the SAR image from that data. However, its characteristic of a reconstructed SAR image has limited performance due to the raw data acquired at the low incident angle.

## 5. Sea Surface Imaging

**Figure 11:**
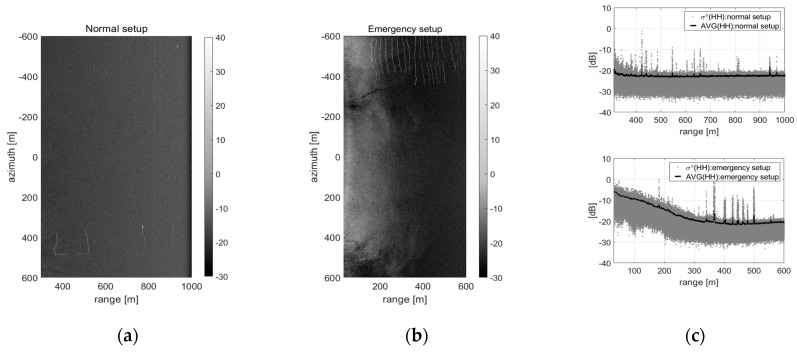
Comparison results of the sea surface SAR image applied the ‘shortened delayed-dechirp process’: on the left side, two images are the SAR images acquired by (**a**) normal setup and (**b**) emergency setup, and (**c**) are their range profiles of signal intensity, respectively.

The sea surface images obtained through each setup for acquiring the raw data of sea clutter can be compared as shown in Figure 11. Figure 11a,b are the bare sea surface SAR images before and after applying the shortened dechirp-delay, and Figure 11c is a comparison result of the signal intensity of each SAR image, respectively. The two images were acquired from adjacent sea areas and included the sea farm buoys. Figure 11a with the normal setup shows a very low level of signal intensity in most of the region of the focused SAR image, while Figure 11b with the emergency setup clearly distinguishes the sea farm buoys and sea surface. The signal characteristics can be easily identified through their range profiles, as shown in Figure 11c. Therefore, it is easily confirmed that the shortened delayed-dechirp process can help to acquire the raw data of sea clutter within a low incident angle and the sea clutter signal is only detectable at the near zone with a low incident angle.

**Figure 12:**
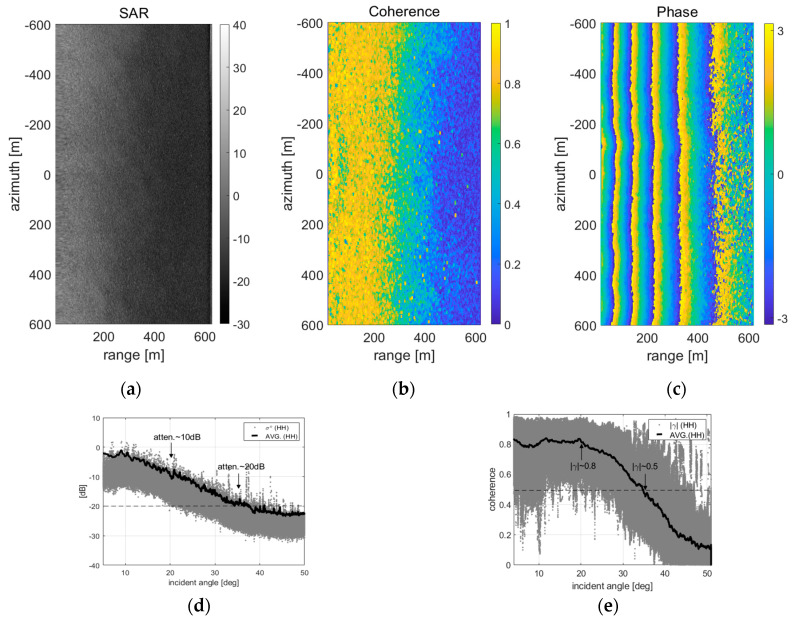
Analysis results of the sea surface SAR image applied ‘shortened delayed-dechirp process’: upper images are (**a**) one of the dual-channel SAR images and (**b**) the coherence and (**c**) phase difference calculated by dual-channel SAR images. Lower graphs are the profiles of (**d**) intensity and (**e**) coherence converted to incident angle.

**Figure 13:**
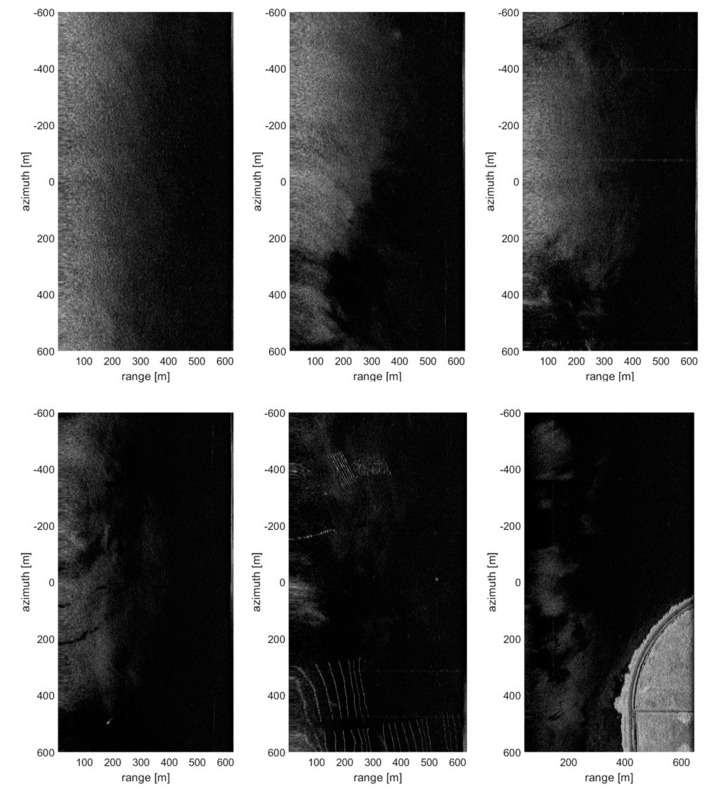
Examples of the reconstructed SAR image for ocean monitoring: upper images show bare sea surfaces without any target, whereas lower images include various targets such as a small ship, sea farm buoys, and shoreline.

The SAR images of the sea surface obtained through the flight experiments applying the shortened delayed-dechirp process are as shown in Figure 12 and Figure 13 (e.g., it is a windy sea surfaces with wind speed 1.3 m/s~3.2 m/s). In particular, Figure 12 is the result of analyzing the signal integrity of the complex SAR images, which are dual-channel SAR images with the configuration of along-track interferometry (ATI). To analyze the signal integrity of the reconstructed SAR images of the sea surface, the dual-channel SAR images and their coherences and phase differences as performance indicators were used. The baseline of 48.26 cm between the master and slave antenna was applied to collect the raw data with ATI configuration and the coherence and phase difference between two images were calculated. In general, the coherence can be used as a measure for the accuracy of the interferometric phase, and the absolute value of coherence, which has a range between 0 and 1, is equivalently described as a function of the signal to noise ratio (SNR) in the standard InSAR process: i.e., |γ| = SNR/(SNR + 1). The coherence between the dual-channel SAR images is calculated by Equation (6) [19]:

(6) $$\left|\gamma \right|=\left|\sum_{n=1}^{N}y_{1}^{\left(n\right)}y_{2}^{*\left(n\right)}\right|/\sqrt{\sum_{n=1}^{N}{\left|y_{1}^{\left(n\right)}\right|}^{2}\sum_{n=1}^{N}{\left|y_{2}^{\left(n\right)}\right|}^{2}},\left(0\leq \left|\gamma \right|\leq 1\right)$$

The SAR image of the sea surface is as shown in Figure 12a, which its size has a swathwidth of about 600 m and a synthetic aperture of about 1.6 km and resolutions are 0.42 m in the range direction and 0.083 m in the azimuth direction, respectively. In order to estimate the coherence from two well reconstructed images (master-/slave-channel), the co-registration of two complex images, which is the first step of standard interferometric SAR processing, was performed. As a result, the coherence and phase difference of the two images can be analyzed. Figure 12b,c shows the coherence and phase difference estimated from the coherent radar signal, respectively. In particular, the phase difference of Figure 12c is the result of the step before removing the cross-track phase component such as the flat earth phase, and it is a preprocessing step in the ATI process for moving target detection or velocity extraction.

Figure 12d is the range profile analyzed from the SAR image of Figure 12a, and the signal attenuation is represented as a function of the incident angle (*θ_i_ = tan^−1^{R_g_/H_0_}*). Figure 12e is likewise represented the coherence of dual-channel images as a function of the incident angle. As shown in Figure 12d, the maximum incidence angle for sea surface imaging can be checked by applying a threshold of about −20 dB, which can be determined by referring to the noise level in the reconstructed SAR image. Only sea clutter signals having a signal level above the noise floor can be reconstructed to the SAR image and it is possible to image the sea surface within the maximum incident angle of about 35°.

It was identified that the maximum incident angle for the sea surface imaging using the ATI system configuration and standard interferometric SAR process is about 35°, which is a little wider than the predicted range of 30° in Figure 7. This result is dependent on the windy sea surface condition of 1.3~3.2 m/s and stable system operation. Additionally, through the analysis result of Figure 12d, it was confirmed that the signal intensity for imaging the sea surface must be higher than the noise floor of about −22.5 dB in the reconstructed SAR image. On the other hand, the sea clutter signal cannot be imaged in the range of more than 40°. As shown in Figure 12c, it is confirmed that the accuracy of the phase component over the range of 400 m is rapidly lowered. Therefore, the optimal incidence angle for imaging the sea surface analyzed based on the above experiment results is a maximum of 35°, referred to as the signal attenuation of 20 dB and coherence of 0.5. This is a value equivalent to about 350 m in terms of ground range (see Table 2).

Figure 13 shows the sea surface SAR images reconstructed with the same experimental settings as above. These sea surface SAR images were reconstructed for the performance check of the emergency setting assuming the maritime observation mission and as input data of the ATI process, they will be used for extracting an ocean current and detecting a moving target. The upper three SAR images are the bare sea surfaces not including any point targets while the lower three SAR images are including a small boat, sea farm buoy, and shoreline. Through the upper three examples, it is confirmed that the sea surface imaging within the ground range of about 350 m or the incident angle of about 35°, which is the same as the previous analysis result, is possible. Moreover, the targets included in the lower three images can be identified in most areas of the SAR image with the low incident angle.

## 6. Conclusions

In this study, a sea surface imaging technique using a ready-made airborne FMCW SAR system for maritime observation on emergency was proposed. The shortened delayed-dechirp process for sea surface imaging was analyzed using observational data and scattering model, and performances of the proposed technique were verified through flight experiments.

The signal attenuation of sea clutter was analyzed using scattering models and measurement data, based on this, the optimal incidence angle range for sea surface imaging could be predicted. The sea clutter signal must keep a higher signal level than the noise floor in the SAR image, and such property was properly reflected in the airborne FMCW SAR system using the shortened delayed-dechirp process. The raw data acquired through the flight experiments were reconstructed to SAR images by the range-doppler algorithm, and the accuracy and stability of the complex data of the SAR image were indirectly measured by coherence and phase difference of the dual-channel SAR images. The maximum incident angle of 35° for the sea surface imaging was confirmed by analyzing the range profile of signal intensity.

Therefore, it was confirmed that the shortened delayed-dechirp process, which was manually readjusted to perform the maritime observation mission, can acquire raw data in a low incidence angle region, and can reconstruct the sea surface SAR image with limited performance and reduced swath width. Moreover, receiving the sea clutter signal within the low incident angle and reconstructing the SAR image can be stably performed using the existing airborne FMCW SAR system. Finally, through this experimental study, it was shown that the existing ready-made FMCW SAR system can also conduct limited missions as an alternative resource for emergency response.

## Figures and Tables

**Table 1:** Specification of an airborne FMCW SAR system.

Radar Parameters	Specifications	Notes
Frequency	10.25 GHz	BW 500 MHz
Tx power	Max 1 W	-
Sampling rate	1.2 MHz	-
Resolution	0.3 m	Slant range
Sensing time	30 s	PRF = 1 kHz
HPBW	11.4°, 37.9°	E-, H-field
Polarization	Horizontal	(HH-pol.)
DAQ	2 channels	Baseline 48.26 cm
Altitude	500 m	-

**Table 2:** Summary of performance comparison (emergency vs. normal setup).

Items	Emergency Setup	Normal Setup	Notes
Dechirp-delay	3.3 μs (*≈2H*_0_*/c*)	3.9 μs	Flight altitude (*H_0_* = 500 m)
Swath width (range)	30~600 m	300~1000 m	
Swath width (angle)	3.5~49°	30~63°	
Max. incidence angle (*θ_i_*)	<35°	-	
Max. range (*R_g_*)	<350 m	-	
Resolution (slant range)	0.5~3.5 m	0.3 m	
Noise floor (in SAR image)	<−22.5 dB	<−22.5dB	
